# Hydrocephalus Following Experimental Subarachnoid Hemorrhage in Rats with Different Aerobic Capacity

**DOI:** 10.3390/ijms22094489

**Published:** 2021-04-26

**Authors:** Yasunori Toyota, Hajime Shishido, Fenghui Ye, Lauren G. Koch, Steven L. Britton, Hugh J. L. Garton, Richard F. Keep, Guohua Xi, Ya Hua

**Affiliations:** 1 Department of Neurosurgery, University of Michigan, Ann Arbor, MI 48109, USA; dyamite2@gmail.com (Y.T.); ranran@med.kagawa-u.ac.jp (H.S.); fenghuiy@umich.edu (F.Y.); hgarton@umich.edu (H.J.L.G.); rkeep@umich.edu (R.F.K.); guohuaxi@umich.edu (G.X.); 2 Department of Physiology & Pharmacology, University of Toledo College of Medicine & Life Sciences, Toledo, OH 43614, USA; Lauren.Koch2@utoledo.edu; 3 Department of Anesthesiology, University of Michigan, Ann Arbor, MI 48109, USA; brittons@umich.edu

**Keywords:** subarachnoid hemorrhage, early brain injury, hydrocephalus, hemeoxgenase-1, CD163, rat

## Abstract

Low aerobic capacity is considered to be a risk factor for stroke, while the mechanisms underlying the phenomenon are still unclear. The current study looked into the impacts of different aerobic capacities on early brain injury in a subarachnoid hemorrhage (SAH) model using rats bred for high and low aerobic capacity (high-capacity runners, HCR; low-capacity runners, LCR). SAH was modeled with endovascular perforation in HCR and LCR rats. Twenty-four hours after SAH, the rats underwent behavioral testing and MRI, and were then euthanized. The brains were used to investigate ventricular wall damage, blood–brain barrier breakdown, oxidative stress, and hemoglobin scavenging. The LCR rats had worse SAH grades (*p* < 0.01), ventricular dilatation (*p* < 0.01), ventricular wall damage (*p* < 0.01), and behavioral scores (*p* < 0.01). The periventricular expression of HO-1 and CD163 was significantly increased in LCR rats (*p* < 0.01 each). CD163-positive cells were co-localized with HO-1-positive cells. The LCR rats had greater early brain injuries than HCR rats. The LCR rats had more serious SAH and extensive ventricular wall damage that evolved more frequently into hydrocephalus. This may reflect changes in iron handling and neuroinflammation.

## 1. Introduction

Subarachnoid hemorrhage (SAH) is a common hemorrhagic stroke that results in striking morbidity and mortality. Early brain injury (EBI) is a concept that indicates overall brain injury, and it might be the primary source of death in patients with SAH [1]. EBI involves elevated intracranial pressure (ICP), blood–brain barrier (BBB) breakdown, and decreased cerebral blood flow, as well as brain edema, oxidative stress, neuronal injury, and death within the acute phase of SAH, usually occurring within 72 h after SAH [1]. SAH patients who died early after hospital admittance (within 72 h) were more likely to present with progressive deficits, seizures, altered consciousness, limb weakness, sensory involvement, and basal ganglia hematoma compared to SAH patients who survived that early period [2]. 

Low exercise capacity is considered to be a risk factor for stroke and many other cardiovascular diseases [3,4,5]. Several studies have associated the incidence of SAH with cardiorespiratory fitness or physical activity. In a study involving a large sample of ethnically diverse patients (*n* = 67,550), good cardiorespiratory fitness was associated with a significantly lower hemorrhagic stroke incidence, including SAH [6]. Another population-based study reported that commuting and leisure-time physical activity may reduce SAH risk in both men and women [7]. With rats bred to gain high and low aerobic capacities, we previously reported that low-capacity runner (LCR) rats exhibited worse brain damage than high-capacity runner (HCR) rats after intracerebral hemorrhage (ICH) [8,9]. In addition, our former study showed that thrombin-induced intraventricular hemorrhage (IVH) caused more intensive hydrocephalus development and periventricular brain damage in LCR rats [10]. However, to our knowledge, no studies have investigated SAH-induced EBI in rats with respect to varied aerobic capacities. In this study, we examined the impacts of different aerobic capacities on early brain injury in a rat SAH model.

Acute hydrocephalus is an important complication after aneurysmal SAH [11], and it is associated with worse outcomes [12]. The current study compared the ventricular changes in LCR rats and HCR rats after SAH in terms of ventricular enlargement, ventricle wall damage, periventricular neuroinflammation, and hemoglobin scavenging.

Neuroinflammation is an important component of cerebral-hemorrhage-induced brain injury, including SAH [13], and we previously found that microglia activation was increased in LCR rats compared to HCR rats after experimental ICH [8]. The haptoglobin (Hp)/CD163 interaction and heme oxygenase-1 (HO-1) signaling pathway are responsible for hemoglobin (Hb) scavenging [14]. The binding of haptoglobin to Hb represents the first defense against Hb neuronal toxicity, which facilitates its elimination by CD163 [15], while the further clearance of Hb includes proteolytic degradation and heme catabolism via the HO-1 signaling pathway. Both CD163 and HO-1 are expressed on microglia and indicate neuroinflammation. The current study investigated rats with varied aerobic capacities in terms of the CD163/HO-1 pathway’s inflammatory response to SAH.

## 2. Results

The baseline aerobic capacity of the HCR and LCR rats was determined. The mean distance (m) run at exhaustion, which was calculated using the measured time (min) and maximal treadmill speed (m/min), was significantly longer in HCR rats than LCR rats (2218 ± 377 m versus 156 ± 39 m, *p* < 0.01). The body weight (g) in LCR rats was significantly greater than in HCR rats (321 ± 28 g versus 241 ± 32 g, *p* < 0.01). Thus, HCR rats performed about 10-fold more work than LCR rats (1333 ± 158 J versus 126 ± 28 J; *p* < 0.01).

**Figure 1:**
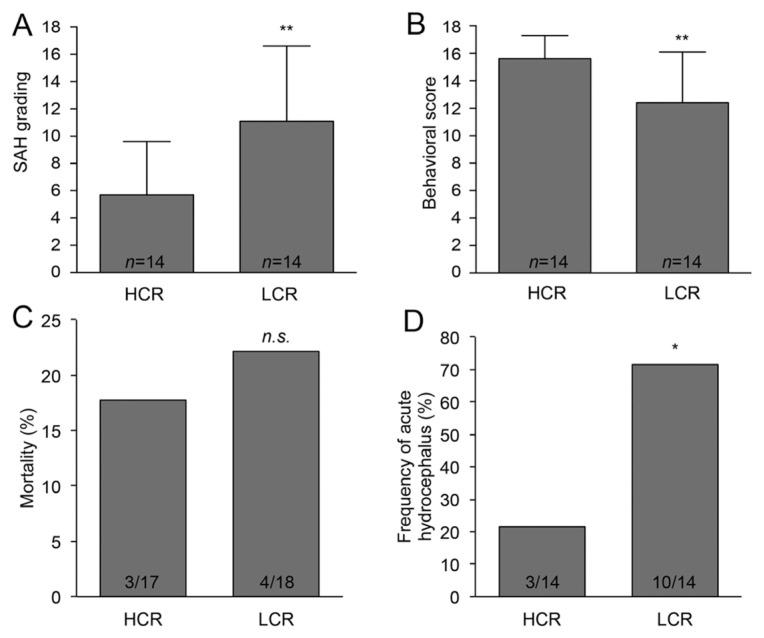
LCR rats had worse SAH grades and behavioral scores, as well as a higher occurrence of hydrocephalus. SAH grades (**A**) and behavioral scores (**B**) in HCR and LCR rats. ** *p* < 0.01 versus HCR group. The % of HCR and LCR rats that died after SAH (**C**) and had acute hydrocephalus 24 h after SAH (**D**); * *p* < 0.05, n.s. (not significant) *p* > 0.05 versus HCR group.

Mortality was determined 24 h after the SAH induction. Neurological functions were evaluated 24 h after SAH with a modified Garcia behavioral test [16], a composite neurological test in which the rats were assessed for various sensorimotor deficits. Immediately after MRI, the rats were euthanized, and the brains were harvested. The intensity of the experimental SAH was evaluated with a modified grading system [17]. The basal brain was divided into six parts, which were each individually assessed with scores from 0 to 3 based on the volume of blood in that region, with the total SAH grade ranging from 0 to 18. LCR rats had significantly higher SAH grading scores (LCR: 11.1 ± 5.5 vs. HCR: 5.7 ± 3.9; *p* < 0.01) and worse behavioral scores (LCR: 12.4 ± 3.7 vs. HCR: 15.6 ± 1.7, *p* < 0.01) compared with HCR rats (Figure 1A,B). There were no neurological deficits in the sham animals. The mortality after SAH was 17.6% (3 of 17 rats) in HCR rats and 22.2% (4 of 18 rats) in LCR rats; there was no difference (*p* > 0.05, Figure 1C). 

**Figure 2:**
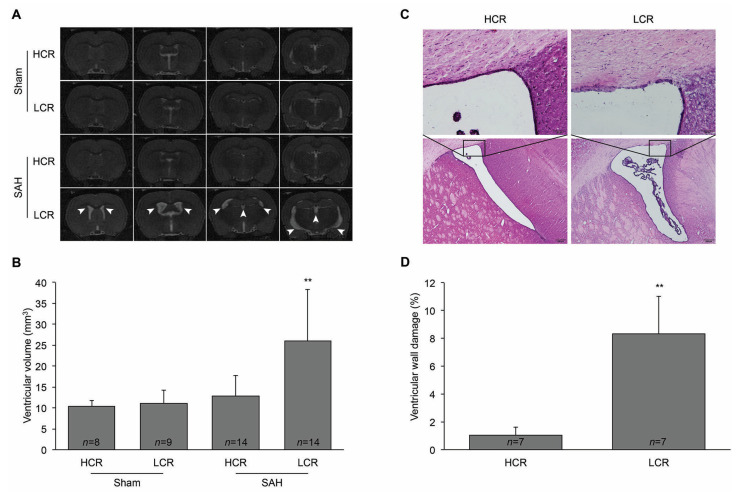
SAH induced hydrocephalus development and ventricular wall damage 24 h after endovascular perforation in LCR rats. (**A**) Representative T2-weighted MRI showing coronal brain section 24 h after SAH or a sham operation in LCR and HCR rats. The hyperintense signal shows brain ventricles. Enlarged ventricles in the LCR rats are indicated by white arrowheads. (**B**) Quantification of the ventricular volume from T2-weighted MRI 24 h after SAH or a sham operation in LCR and HCR rats. (**C**) Representative hematoxylin and eosin staining of brain sections showing ventricular wall damage 24 h after SAH or a sham operation in LCR and HCR rats. The top micrographs are enlargements of the boxed areas in the lower micrographs. Scale bars: top, 50 μm; bottom, 200 μm. (**D**) Quantification of ventricular wall damage 24 h after SAH or a sham operation (percentage of the damaged ventricular wall in the total ventricle wall length). Values are mean ± SD; ** *p* < 0.01 versus HCR group.

Hydrocephalus was determined when the ventricular volume increased more than +3 SD above the mean in sham rats. The frequency of hydrocephalus was significantly greater in LCR (71%; 10/14) than in HCR rats (21%; 3/14; *p* < 0.05, Figure 1D). In LCR rats, SAH induced apparent ventricular enlargement at 24 h (26.0 ± 12.3 mm^3^; *n* = 14) compared with HCR rats (12.8 ± 4.9 mm^3^; *n* = 14; *p* < 0.01, Figure 2A,B). In addition, the percentage of damaged ventricular wall was higher in LCR rats (8.3 ± 2.7%; *n* = 7) than in HCR rats (1.03 ± 0.6%; *n* = 7; *p* < 0.01, Figure 2C,D).

**Figure 3:**
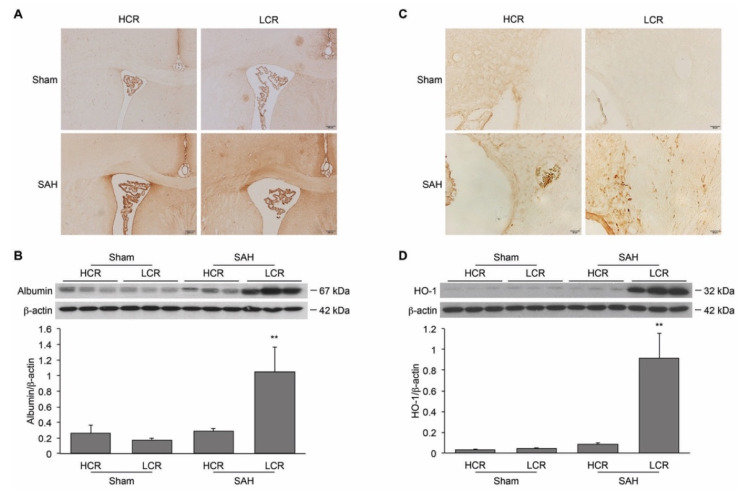
SAH induced greater BBB breakdown and more HO-1 upregulation 24 h after SAH in LCR rats than in HCR rats. (**A**) Representative images of periventricular albumin immunohistochemistry in LCR and HCR rats after SAH or a sham operation. Albumin-positive staining is indicated by the darker color in brown intensity. Scale bar = 200 μm. (**B**) Albumin levels were quantified by Western blotting. Values are mean ± SD; ** *p* < 0.01 LCR + SAH versus HCR + SAH. (**C**) Representative images of periventricular HO-1 immunohistochemistry in LCR and HCR rats after SAH or a sham operation. Scale bar = 20 μm. (**D**) HO-1 levels were quantified by Western blotting. Values are mean ± SD; ** *p* < 0.01 LCR + SAH versus HCR + SAH.

The BBB breakdown in LCR and HCR rats was examined by measuring albumin extravasation in the periventricular region. Immunohistochemistry staining revealed greater albumin extravasation after SAH in LCR rats than in HCR rats (Figure 3A), which was confirmed with a Western blotting analysis (LCR albumin/β-actin ratio 1.04 ± 0.32 vs. 0.29 ± 0.04 in HCR rats; *p* < 0.01, Figure 3B). 

HO-1 is a stress protein involved in heme degradation. Immunohistochemistry staining revealed increased periventricular HO-1-positive cells in LCR compared to HCR rats 24 h after SAH (Figure 3C). In addition, Western blotting showed that the protein levels of HO-1 were higher in SAH LCR rats (ratio to β-actin: 0.92 ± 0.24) compared to SAH HCR rats (0.086 ± 0.009) and sham controls at 24 h (0.032 ± 0.007 in HCR rats, 0.047 ± 0.006 in LCRs; *p* < 0.01, Figure 3D).

**Figure 4:**
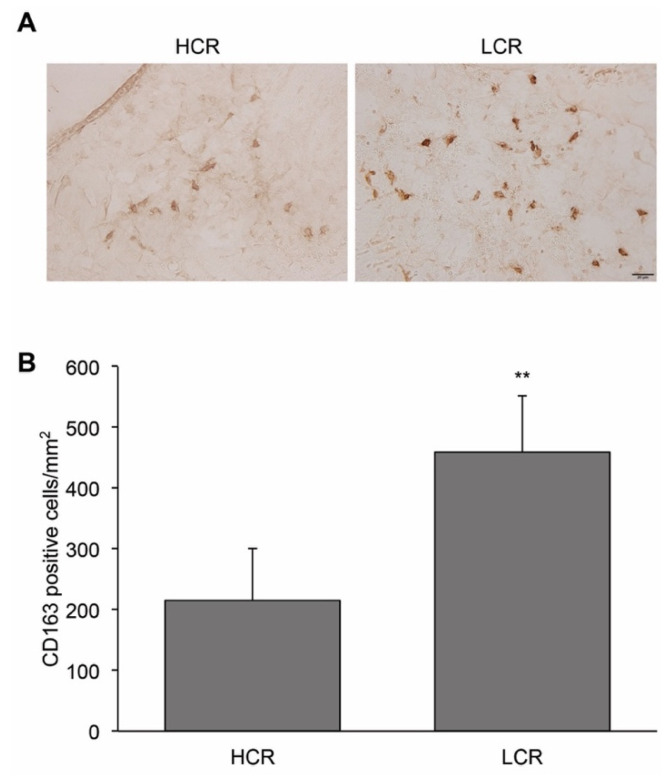
The number of CD163-positive cells after SAH was greater in LCR rats than in HCR rats. (**A**) Representative images of periventricular CD163 immunohistochemistry 24 h after SAH in HCR and LCR rats. Scale bar = 20 μm. (**B**) Quantification of the number of CD163-positive cells after SAH. Values are mean ± SD; ** *p* < 0.01 versus HCR group.

CD163 is a hemoglobin scavenger receptor. No CD163 positive cells were detected in the periventricular areas of the sham groups. In the SAH groups, the number of periventricular CD163-positive cells was significantly greater in LCR rats (459 ± 94 cells/mm^2^) than in HCR rats (216 ± 84 cells/mm^2^; *p* < 0.01, Figure 4A,B).

## 3. Discussion

In the present study, the major findings were: (1) The endovascular perforation model for SAH resulted in greater hemorrhage volume, more severe ventricular enlargement, worse behavioral outcomes, and increased BBB breakdown in LCR rats compared to HCR rats, and (2) periventricular CD163 and HO-1 expression was also greater in LCR rats. These results indicate a significant impact of exercise capacity on the acute pathological changes following SAH.

The LCR–HCR model system was established to investigate the impact of aerobic capacity on disease risk. It provides a polygenic-based experimental substrate that can explore both diagnostic and prescriptive mechanisms that underlie diseases and risk factors. It is widely considered that low exercise capacity is a risk factor for hemorrhagic and ischemic stroke [4]. Our previous studies showed differences in animal models of ICH- and IVH-induced brain injury between varied aerobic capacities [8,9,10]. However, no studies have focused on SAH-induced brain injury in rats with different aerobic capacities. In this study, we found that LCR rats had a greater hemorrhage volume after experimental SAH than HCR rats. Further, LCR rats developed more significant ventricular enlargement than HCR rats, and they had more severe neurological deficits and worse BBB breakdown. SAH volume in patients is correlated with higher blood pressure [18], and the LCR rats had higher blood pressures than those of HCR rats (mean arterial pressure 102 vs. 90 mmHg) [19]. However, there may be other contributory factors, including differences in the coagulation cascade. In humans, there is evidence that exercise impacts coagulation factors [20,21]. Further studies concerning the differences in SAH volume are needed. While it is possible that the worse outcomes after SAH in LCR rats might just reflect differences in subarachnoid hemorrhage volume, it should also be noted that in other hemorrhage-related models where the insult was fixed (amount of blood injected intracerebrally or the dose of thrombin or iron injected), the brain injury in LCR rats was considerably worse compared to that in HCR rats [8,9,10]. 

Acute hydrocephalus is a common complication of SAH and is an indicator of worse outcomes [12]. The mechanisms of post-hemorrhagic hydrocephalus were thought to involve obstruction of cerebrospinal fluid (CSF) outflow pathways by blood components and subarachnoid fibrosis, all leading to impaired CSF outflow. However, ependymal damage and dysfunctional cilia may also contribute to hydrocephalus development [22]; in the current study, ventricular wall damage was more severe in LCR rats than in HCR rats.

The periventricular level of albumin, which is an indicator of BBB breakdown, was markedly increased after SAH in LCR rats. BBB breakdown and brain edema are major components in EBI after SAH. Multiple mechanisms have been suggested to underlie SAH-induced BBB breakdown, including initial cerebral ischemia, the effects of thrombin and iron from the clot, and inflammatory mediators, including cytokines, chemokines, and matrix metalloproteinases [23,24,25]. In addition, hydrocephalus affects BBB breakdown due to oxidative stress [26]. 

The Hp/CD163 and HO-1 signaling pathways are known as an efficient captor–receptor–enzyme system that plays an important role in eliminating the Hb-/heme-induced toxicity after hemolysis [15]. CD163 is highly expressed in microglia/macrophages after being activated toward M2/phagocytic microglia/macrophages, where it acts as a hemoglobin scavenger receptor that participates in the removal and endocytosis of Hp–Hb complexes [27,28,29]. CD163-mediated Hp–Hb is transferred to early endosomes and subsequently degraded into heme [15]. Heme is then metabolized by HO-1(also referred to as heat shock protein 32), a stress-induced protein, into carbon monoxide, ferrous iron, and biliverdin. The current study showed that SAH caused greater periventricular upregulation of CD163 and HO-1 in LCR rats compared with HCR rats. Although it is possible that this greater upregulation might just reflect more subarachnoid bleeding, there is evidence to suggest that this may not be the sole cause. Thus, in an ICH model with a set volume of intracerebral blood, LCR rats also had a greater perihematomal HO-1 upregulation [8]. 

The roles that CD163 and HO-1 play in brain injury are complex. After ICH, CD163 knockout mice initially have less brain injury than wild-type controls, but after 4 days, they have worse injuries [29]. The underlying mechanisms for this switch are still uncertain, but those results suggest that the greater CD163 expression in LCR rats early (one day) after SAH may be detrimental. That adverse effect might reflect the uptake of hemoglobin and the release of iron in neurons, as well as microglia [30,31]. Similarly, there is evidence that while HO-1 may lead to iron overload and result in brain injury after SAH and ICH [32,33], a recently published study also showed that HO-1 expression in microglia was related to neuroprotection after SAH [34]. HO-1 has diverse physiological and pathological functions that might depend on the cell types, injury stimuli, and time [35]. Whether the increased expression of HO-1 in LCR rats after SAH is protective or detrimental is, as yet, uncertain. However, the increased expression suggests increased cell stress.

This study, along with other investigations looking into the aerobic capacity with SAH [6,7] or aerobic exercise after SAH [36,37], opens new and encouraging perspectives for early brain injury and protection following SAH. Understanding the pathways affected by aerobic capacity that impact SAH may allow pharmacological manipulation to limit SAH-induced injury. Future prospective randomized human studies on the impact of aerobic capacity are warranted for more evidence and provide a new therapeutic strategy for SAH.

## 4. Materials and Methods

### 4.1. The Animal Preparation and SAH Induction

The animal-use protocols were approved (PRO00006054, 5 February 2015) by the Institutional Animal Care and Use Committee (IACUC) at the University of Michigan. Animals were housed under a standard 12–12 h light–dark circle and guaranteed adequate water and food supply. A total of 25 adult male HCR rats and 27 adult male LCR rats (5–6 months old, generation 32) were used in this study. The details on selectively breeding rats of different aerobic capacities were described previously [19]. Experimental SAH was induced with the endovascular perforation technique, as described previously [17]. Briefly, rats were anesthetized with isoflurane (5% induction) and placed on a controlled heated pad to maintain core body temperature at 37.5 °C. Mechanical ventilation was initiated after proper intubation, and isoflurane was then controlled between 2.5 and 3%. A middle skin incision was made for the visual exposure of the common carotid artery. Following sectioning, the external carotid artery, a rounded 3-0 monofilament suture, was introduced into the internal carotid artery and carefully advanced along until resistance was felt. Then, the surgeon slightly pushed the suture further to create a perforation on the artery. After that, the suture was gently withdrawn to produce SAH, and the skin was sutured. The sham animals went through the same operation, except for the perforation part (HCR; *n* = 8, LCR; *n* = 9). 

Twenty-four hours after the SAH or sham operation, all rats underwent behavioral testing and magnetic resonance imaging (MRI) scanning. The rats were then euthanized with a lethal dose of pentobarbital and perfused with 4% paraformaldehyde. SAH grading was done after the removal of the brains, which were further processed for histology and immunohistochemistry.

### 4.2. MRI Investigation 

Rats were anesthetized with a 1.5% isoflurane and air mixture during the scanning. T2-weighted imaging was performed in a 7.0 T Varian MR scanner (Palo Alto, CA, USA) with a field of view of 35 × 35 mm, and a total of 25 coronal slices were obtained. Ventricular volume was analyzed as reported previously [32]. Briefly, in all MRI slices, ventricles, including the lateral ventricles, the third ventricle, and the aqueduct, were outlined and measured, then summed up and multiplied by section thickness (0.5 mm). MRI evaluation was done with ImageJ software (NIH) by a masked investigator.

### 4.3. SAH Grading

The intensity of SAH was evaluated by a modified grading system as described earlier [17]. In general, the basal brain, including the brain stem, was divided into six parts to be evaluated individually from 0 to 3 based on the volume of blood, with the total SAH grade ranging from 0 to 18.

### 4.4. Behavioral Tests

Neurological functions were evaluated 24 h after SAH with a modified Garcia behavioral test [16]. The neurobehavioral test consisted of six subtests: 1. spontaneous activity; 2. symmetry in the movement of four limbs; 3. forepaw outstretching; 4. climbing; 5. body sensation; 6. vibrissae touch. The minimum neurological score was 2 and the maximum was 18.

### 4.5. Ventricular Wall Damage Evaluation

Ventricular wall damage was evaluated as described earlier [32]. Briefly, for each rat, sections of three layers of the ventricular system were stained by hematoxylin and eosin. The first layer, around bregma −0.4 mm at the sagittal midline, showed a bilateral frontal ventricular horn. The second layer, around bregma −1.2 mm, demonstrated the bilateral lateral ventricle and its connection with the anterior third ventricle. The third layer, around bregma −4.0 mm, revealed posterior bilateral lateral ventricles and the body of the third ventricle. The ventricular wall in each layer was inspected for any discontinuities, breakdown, or detachment of the ependyma layer from the periventricular parenchyma. The ventricular wall damage extent was presented by the percentage of the damaged wall compared to the total length of the ventricular wall. All analyses were performed with the ImageJ software (NIH, Bethesda, MD, USA) by a masked investigator.

### 4.6. Immunohistochemistry

The brains were immersed in 4% paraformaldehyde before transfer to a 30% sucrose in 0.1 M cold phosphate-buffered saline (pH 7.4) solution for 2–3 days at 4 °C to avoid crystal formation. The brains were embedded before sectioning coronally to 18 μm on a cryostat. Immunohistochemistry was performed with the avidin–biotin complex technique. Sections were blocked by 1:10 goat or horse serum (Vector Laboratories,Burlingame, CA, USA) at room temperature for 30 min and then incubated at 4 °C overnight with the primary antibody. The primary antibodies were polyclonal rabbit anti-HO-1 (1:400 dilution, Enzo, Farmingdale, NY, USA), monoclonal mouse anti-rat CD163 (1:50 dilution, Bio-Rad, Hercules, CA, USA), and polyclonal sheep anti-rat albumin (1:4000 dilution, Bethyl Laboratories, Montgomery, TX, USA). The secondary antibodies included goat anti-rabbit IgG (1:500 dilution, Bio-Rad, Hercules, CA, USA), horse anti-mouse IgG (1:500 dilution, Bio-Rad, Hercules, CA, USA), and horse anti-sheep IgG (1:500 dilution, Bio-Rad, Hercules, CA, USA). Negative controls were obtained by the omission of the primary antibody. 

### 4.7. Western Blotting Analysis

The Western blotting analysis was performed as reported previously [38]. Briefly, periventricular or cortical tissues were collected and sonicated in Western blotting sample buffer. Protein concentration was detected by the Bio-Rad protein assay kit, and 40 μg protein samples were loaded and then separated by sodium dodecyl sulfate–polyacrylamide gel electrophoresis before transfer to a Hybond-C pure nitrocellulose membrane (Amersham, St. Louis, MO, USA). Membranes were probed with the following primary antibodies: polyclonal rabbit anti-HO-1 (1:2000 dilution, Enzo, Farmingdale, NY, USA) and polyclonal sheep anti-rat albumin (1:10,000 dilution, Bethyl Laboratories, Montgomery, TX, USA). Antigen–antibody complexes were visualized with the ECL technique. Image analysis was done with the ImageJ software by a masked investigator.

### 4.8. Cell Counting

High-power images (×40 magnification) taken in the periventricular area by a digital camera with sections 0.5 mm anterior to bregma were used to calculate the number of immuno-positive cells. The number of CD163-positive cells in three square images per section was counted and statistically analyzed with the ImageJ software by a masked investigator.

### 4.9. Statistical Analysis

Unless stated, values are presented as means ± SD. Statistical differences among groups were analyzed using Student’s *t*-test, a chi-square test, or one-way ANOVA with a Tukey post hoc test. Statistical significance was set at *p* < 0.05. Graphpad Prism (San Diego, CA, USA) was used to analyze the data.

## 5. Conclusions

In summary, rats with low aerobic capacity exhibit more profound early brain injury following SAH compared to those with high aerobic capacity. Rats with low aerobic capacity had greater SAH volumes, ventricular enlargement, and BBB breakdown, as well as worse neurological outcomes. These elevated risk factors were associated with alterations in hemoglobin-handling proteins and inflammatory mediators. Understanding the mechanisms by which aerobic capacity affects SAH-induced injury may provide therapeutic opportunities for this devastating form of stroke.

## Data Availability

All supporting data are available within the article.

## Abbreviations

SAH	subarachnoid hemorrhage
HCR	high-capacity runners
LCR	low-capacity runners
HO-1	heme oxgenase-1
EBI	early brain injury
ICP	intracranial pressure
BBB	blood–brain barrier
IVH	intraventricular hemorrhage
Hp	haptoglobin
Hb	hemoglobin
CSF	cerebrospinal fluid
